# Geochemical and Geophysical Monitoring of Hydrocarbon Seepage in the Adriatic Sea †

**DOI:** 10.3390/s20051504

**Published:** 2020-03-09

**Authors:** Marzia Rovere, Alessandra Mercorella, Emanuela Frapiccini, Valerio Funari, Federico Spagnoli, Claudio Pellegrini, Andree Soledad Bonetti, Tiziana Veneruso, Anna Nora Tassetti, Marcello Dell’Orso, Marco Mastroianni, Giordano Giuliani, Rocco De Marco, Gianna Fabi, Francesco Ciccone, Ilaria Antoncecchi

**Affiliations:** 1 Istituto di Scienze Marine, National Research Council, Via P. Gobetti 101, 40129 Bologna, Italy; alessandra.mercorella@bo.ismar.cnr.it (A.M.); valerio.funari@bo.ismar.cnr.it (V.F.); claudio.pellegrini@bo.ismar.cnr.it (C.P.); f.ciccone.ext@mise.gov.it (F.C.); 2 Istituto per le Risorse Biologiche e le Biotecnologie Marine, National Research Council, Largo Fiera della Pesca 2, 60125 Ancona, Italy; emanuela.frapiccini@cnr.it (E.F.); federico.spagnoli@cnr.it (F.S.); annanora.tassetti@cnr.it (A.N.T.); giordano.giuliani@cnr.it (G.G.); r.demarco@irbim.cnr.it (R.D.M.); gianna.fabi@cnr.it (G.F.); 3 Dipartimento di Scienze della Vita e dell’Ambiente, Università Politecnica delle Marche, Via Brecce Bianche, 60121 Ancona, Italy; 4 DGS UNMIG Division V, Ministry of Economic Development, Via A. Bosio 13B, 00161 Roma, Italy; andreesoledad.bonetti@mise.gov.it (A.S.B.); tiziana.veneruso.ext@mise.gov.it (T.V.); marcello.dellorso@mise.gov.it (M.D.); marco.mastroianni@mise.gov.it (M.M.); ilaria.antoncecchi.ext@mise.gov.it (I.A.); 5 Ricerca sul Sistema Energetico, Via Rubattino 54, 20134 Milano, Italy

**Keywords:** Mediterranean Sea, biogenic methane, benthic chamber, pockmarks, geochemical proxy, cold seep, hydrocarbon exploration

## Abstract

Hydrocarbon seepage is overlooked in the marine environment, mostly due to the lack of high-resolution exploration data. This contribution is about the set-up of a relocatable and cost-effective monitoring system, which was tested on two seepages in the Central Adriatic Sea. The two case studies are an oil spill at a water depth of 10 m and scattered biogenic methane seeps at a water depth of 84 m. Gas plumes in the water column were detected with a multibeam system, tightened to sub-seafloor seismic reflection data. Dissolved benthic fluxes of nutrients, metals and Dissolved Inorganic Carbon (DIC) were measured by in situ deployment of a benthic chamber, which was used also for the first time to collect water samples for hydrocarbons characterization. In addition, the concentration of polycyclic aromatic hydrocarbons, as well as major and trace elements were analyzed to provide an estimate of hydrocarbon contamination in the surrounding sediment and to make further inferences on the petroleum system.

## 1. Introduction

Hydrocarbon seepages are often found on the seafloor both in shelfal and deeper marine environments [1,2]. Hydrocarbons are organic compounds that contain only carbon and hydrogen atoms. In nature, hydrocarbons, both as gaseous (methane C1, ethane C2, propane C3, butane C4) and liquid phases (benzene, hexane, octane, C5+), are mainly found in the porosity of the rocks that make up the upper continental crust of the Earth and are the product of chemical-physical processes that may persist for thousands to millions of years. Hydrocarbons having lower molecular weight are found in the gaseous state, while those with higher molecular weight are liquid or waxy solids.

Hydrocarbons are less dense than the surrounding rocks and sediments, thus they tend to migrate to shallower sedimentary horizons and eventually to “pierce” the seabed, giving rise to peculiar seafloor features, such as pockmarks [3] and mud volcanoes [4]. This phenomenon was first discovered by Alessandro Volta, who in 1776 noted that flammable gas bubbles were forming when shaking the muddy bottom of Lake Maggiore and called it “Gas di Palude” (swamp gas). In 1600, French explorers observed some Native Americans developing fires on the surface of Lake Erie in North America. In the deep ocean, hydrocarbon seeps were first discovered along the continental slope of Florida [5] and Louisiana [6] in the Gulf of Mexico in the early 1980s.

Marine environments are ideal for the formation of hydrocarbons because organic matter (e.g., phytoplankton, zooplankton, terrestrial and marine plants) settles in large quantity on the sea bottom and rapidly undergoes anaerobic degradation due to anoxic conditions. In addition, high and fast sedimentation rates, typical of certain marine depositional environments, favour the rapid burial and decomposition of organic matter, due to increasing temperature with depth. Methane is by far the most common gas in the sedimentary rocks of the Earth’s crust and deep marine sediments are the largest reservoir of methane, mostly in their hydrate form (104 Gt of C [7]). Some estimates indicate that various sources of methane annually inflow into the oceans 0.02 Gt of C, although most is thought to sink in the carbonate precipitation-related processes [8]. Hydrocarbon generation can be either a microbic process mediated by specialized bacterial communities living in anoxic sediments at <50 °C [9] and <1 km from the seabed [10] or a thermic decomposition of the organic matter, normally at temperatures exceeding 150–160 °C, at deeper depths [9]. 

When the flow is sufficiently high, methane can escape the seabed and form gas plumes in the water column. These can be detected as density anomalies by marine geophysical instruments, such as echo sounders, side scan sonar [11] or multibeam systems [12]. Direct gas sampling in aquatic environments is not as straightforward as in terrestrial cases, due to the high hydrostatic pressures involved and the need to keep the sampled fluids at their native pressure. In shallow waters the employments of scuba divers may be envisaged, but the recommended maximum depth for conventional scuba diving is 40 m and even less (30 m) under the European scientific diving standards [13]. While in waters <30 m, operational costs may be kept down using relatively simple and inexpensive samplers such as “vacuum” containers to be manually operated [14], in deeper marine environments, more sophisticated isobaric gas-tight samplers [15] have to be operated through very expensive remote operated vehicles (ROVs).

**Figure 1:**
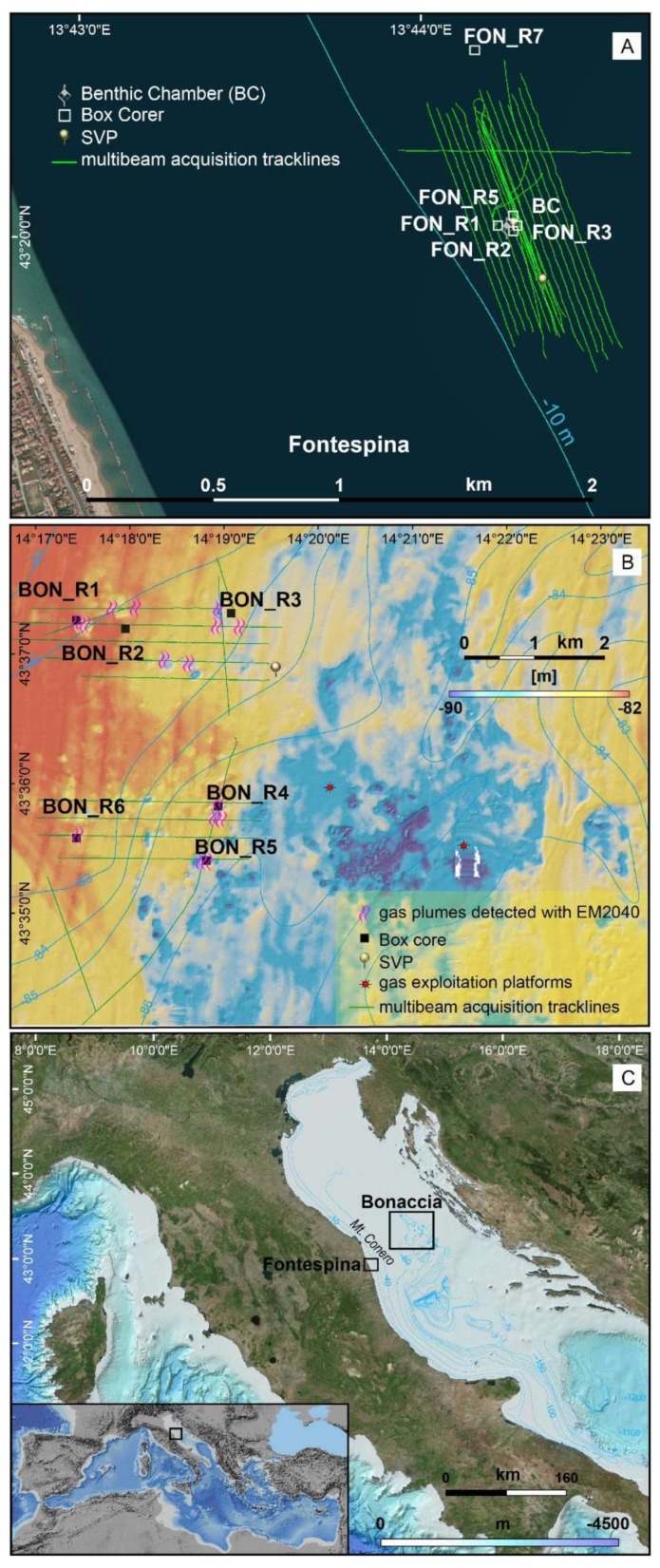
The Fontespina (**A**) and Bonaccia (**B**) study areas and sampling sites in the Central Adriatic Sea (**C**).

This contribution regards the setup of a new cost-effective and relocatable integrated monitoring system able to detect and monitor offshore hydrocarbon seepage and water column plumes of natural or anthropic origin, by means of techniques and know-how developed by CNR and the Ministry of Economic Development of Italy and adapted from other uses [16]. We decided to test this new integrated system in shallow and intermediate marine environments, where organic matter sinks to the sea bottom and undergoes rapid burial and anaerobic degradation, becoming deeply-trapped. For this reason, two sites have been investigated in the Central Adriatic Sea: one is an oil spill, known since the 1940s, located at shallow water depth of 10 m about 1 mile off Fontespina village (Fontespina site, FON in tables and figures) that was previously sampled for geochemical gas composition [17] and ecotoxicological assessment of seepage on caged eels and mussels [18]. The second study area is located about 60 km offshore Mt. Conero, at water depth of 84 m nearby a cluster of biogenic methane gas exploitation plants (Bonaccia site, BON in tables and figures) (Figure 1). The name “Bonaccia”, which in Italian refers to calm sea state, was chosen in response to the usually harsh wind conditions of this stretch of sea. This area was previously investigated by geophysical and geological studies [19,20,21]. 

The overarching aim of this kind of studies is to determine and understand the transport processes of hydrocarbon gas from the sub-seabed to the seabed and the water column and potentially to the atmosphere, where they might increase the global carbon budget. Furthermore, understanding the migration of hydrocarbons in the subsurface is of primary importance for oil and gas exploration, because subtle features of fluid migration are often overlooked on large-scale seismic reflection data though may have important implications in the reservoir characterization. Finally, monitoring natural or human-induced gas seepage is important to mitigate the adverse effects of hydrocarbon spill and discharge, especially if located near the coast or nearby human activities. In this contribution, we address some of the above mentioned key points, starting from the results of the survey conducted to test a sampling methodology.

## 2. Materials and Methods

High-resolution bathymetric data of the Bonaccia area were previously acquired in 2012, using the hull-mounted Kongsberg EM710 (70–100 kHz) on board R/V Urania [19]. Sound velocity profiles were calculated from a Sea-Bird SBE 911 PLUS Conductivity-Temperature-Depth (CTD) profiler. Multibeam data have been merged and post-processed using the software CARIS HIPS & SIPS (version 11.0.8) to produce a 10 m resolution DTM over the entire area. 

Sub-bottom seismic profiles were acquired on board R/V Urania in 2012, with a Teledyne Benthos CHIRP-III system, comprised of a 16 hull-mounted transducer array and using a 2–20 kHz sweep-modulated bandwidth and 4 kW power per-channel, which allows a vertical resolution of about 50 cm. During the same survey, a multichannel seismic reflection line was acquired using a mini water-gun source (Sercel S15-02 of 15 inc^3^) with a 80–500 Hz frequency band width and a shot interval of 3.125 m and recorded through a Teledyne mini-streamer with 24 channels and group interval of 3.125 m. Sampling rate was 0.25 ms, with a record length of 832 ms TWT (Two Ways Time). Traces were processed using the software Disco/Focus by Paradigm^®^ up to time migration. Normal Move Out correction was performed using a simplified velocity model with sound velocity increasing from 1500 m s^−1^ at the seabed increasing to 1850 m s^−1^ at 0.832 s. Single-channel sparker profiles were acquired with a Geo-Spark 1000 (1 kJ) source and recorded with a towed Edgetech 265 hydrophone on board R/V Minerva Uno in 2015.

The monitoring pilot test, which represents the core acquisition of this study, has been carried out on board the R/V Tecnopesca II, in August 2018. During the survey three kinds of data have been acquired: acoustic backscatter of the water column by a multibeam sonar system; water samples and dissolved benthic fluxes at the sediment-water interface, measured by an automatic benthic chamber; sediment samples collected nearby seepage sites by a box corer for geochemical laboratory analyses.

A hull-mounted multibeam dual-head Kongsberg EM2040 CD was used to detect and record gas plumes in the water column. The EM2040 system has a large swath coverage (140–200°) with operating frequency range 200–400 kHz. For this specific water column survey, an optimal frequency between 250 and 300 kHz was used. Sound velocity profiles were obtained with an AML Smart Sound Velocity and Pressure (SV & P) sensor and were applied in post-processing. Bathymetric and seafloor reflectivity data were post-processed using the suite CARIS HIPS & SIPS, while QPS Fledermaus (version 7.8, including the FMMidwater module) and Pty Ltd. Echoview^®^, Tasmania (version 10.0) software packages were used to acquire and investigate different information simultaneously: water column reflectivity data, positions of target seepages, and volumes extraction of gaseous plumes in 3D mode. 

**Figure 2:**
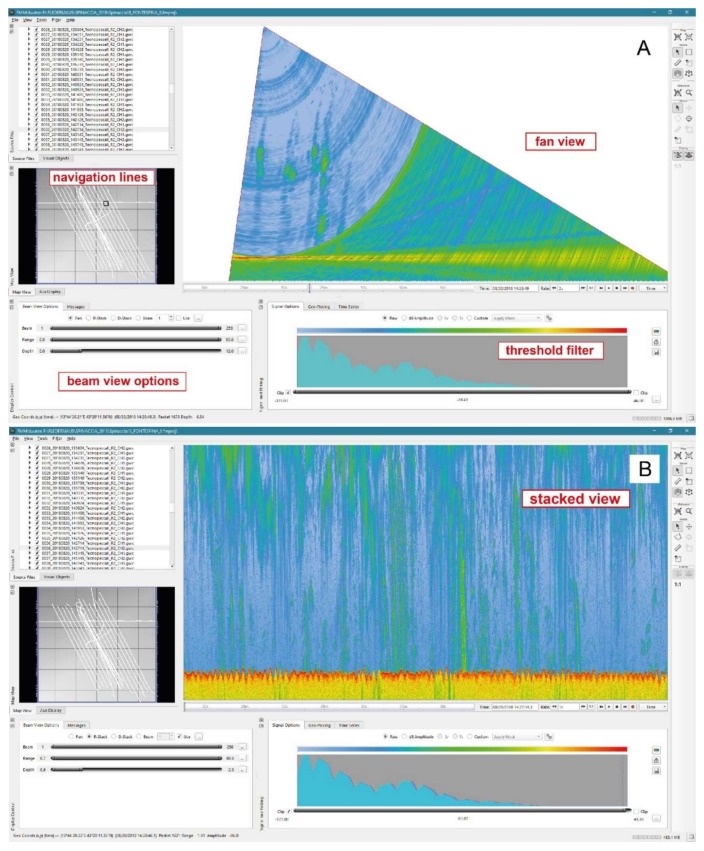
Acoustic anomalies associated with natural seeps observed in the FMMidwater Fan View (**A**) and in Stacked View (**B**). The gas plumes rising sub-vertically from the seafloor are visible without applying a filter.

Native Kongsberg. wcd data were converted into a GWC (generic water column) format and imported in the QPS FMMidwater module, which provides multiple ways to display the data, allowing easy and rapid manual identification of features for selection, thresholding and extraction of data. The fan view is a traditional along-track view of the water column data with the viewpoint from astern and looking towards the bow and underwater; a time bar control allows to move along the acquired line (Figure 2A). The stacked view, instead, allows to view all time-series information from all currently selected beams ‘stacked’ on top of each other. This view preserves target geometry and uses/displays the maximum signal level at a given time-based pixel. In this view, a status bar updates the range and signal amplitude at the specific location (Figure 2B).

**Figure 3:**
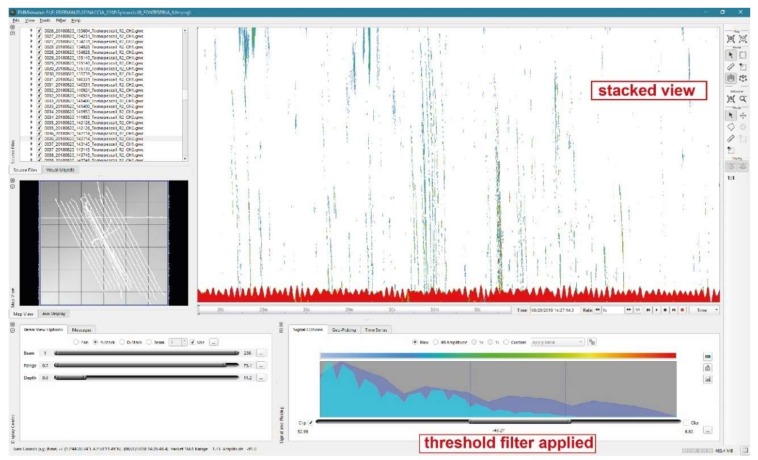
Manually filtered plumes in the FMMidwater Stacked View with background noise deleted.

The subsequent application of the normalization filter generates background values (dB) made up of the persisting features (i.e., the deep scattering layer, seafloor, water column reverberation). If the background is considered to be the ‘noise’ against which the targets are detected, then setting the threshold is equivalent to set a fixed signal-to-noise level threshold. The normalization filter generates a moving average of the ‘background’ amplitude/dB value over a specified window size (based on a manually selected number of pings in the GWC file) and sets a threshold based on that value. The next step of the process involves adjusting thresholds on normalized data. In this way, it is possible to focus on specific water column features, such as gas plumes, in order to highlight and export them (Figure 3).

Finally, water column features were manually extracted and exported as Refracted Points (ASCII), which include longitude, latitude, depth and signal value corrected with sound velocity profiles and suitable for a further editing/cleaning in Fledermaus or any other software. 

**Figure 4:**
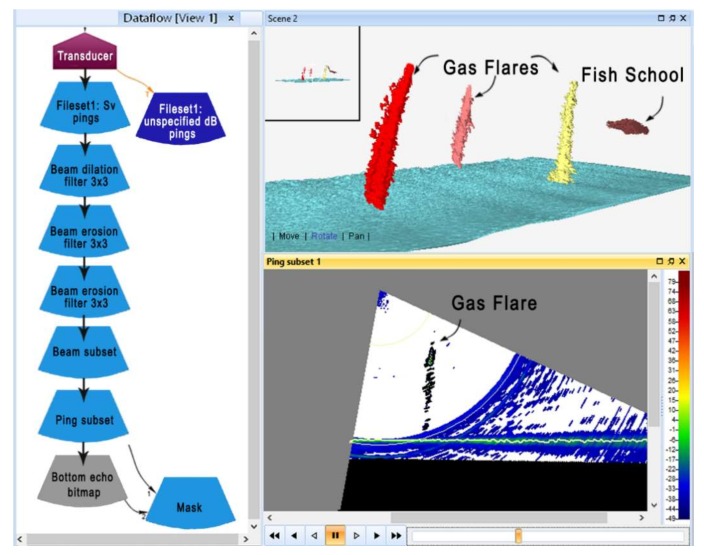
Standard Echoview^®^ Dataflow Window showing the operators used for automatically filtering the data (**left**); the 3D scene window with the cluster regions detected in the ping subset (**upper right**); the ping subset window of the filtered multibeam echogram displaying a gas flare in the water column (**lower right**).

A similar dataflow was used in Echoview^®^ for gas seeps’ detection, even if additional operators/filters were applied to perform semi-automated and objective processing of high volumes of data. In order to make target detection easier, multibeam data were smoothed using different operators to reduce data gaps and a minimum threshold was set to remove noise while maintaining useful backscatter data. The data-processing workflow was designed in the Dataflow Window (Figure 4) to strike a balance between the initial quality of the multibeam data, processing goals (locating and describing plume signals) and efficiency. Ping sub setting data and dataflow adjustments were sometimes necessary to refine results and limit target loss.

Finally, the school detection algorithm was used to clump together separate water column features, that were next to each other, into single cluster regions (e.g., gas flares and fish schools), to exclude the acoustic sea bottom and apply a morphological filter that removes strong acoustic signals that do not conform to the expected shape of a gas plume (linear and mostly vertical), and that in our case mostly corresponded to fish schools. Cluster regions can be viewed in 3D scenes (Figure 4).

**Figure 5:**
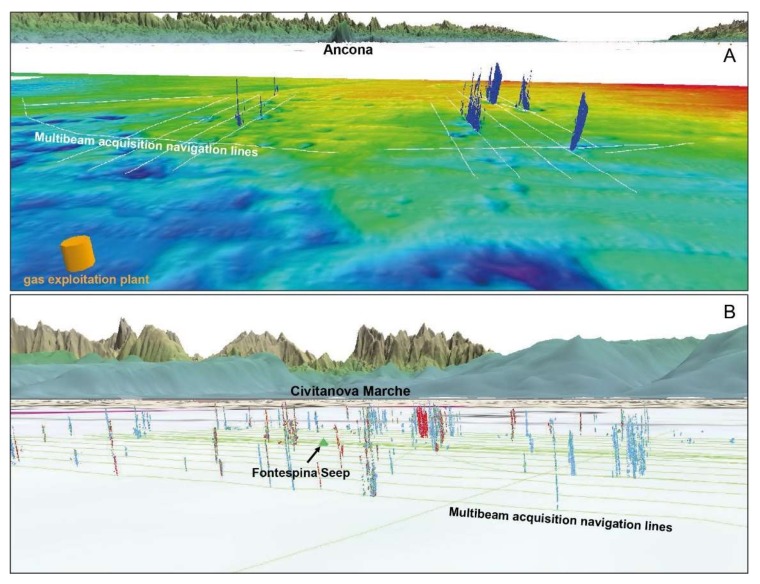
3D visualization (Fledermaus scene) of processed gas plumes in the water column at the Fontespina site (**A**) and Bonaccia site (**B**). Each point’s colour may variably represent plumes’ height in the water column, amplitude or number of beams.

Once detected, georeferenced seep data were imported as points into Fledermaus software to give overall 3D views of seafloor signatures associated with gas plumes and additional object models. In particular, Fledermaus allowed to assemble and explore virtual 3D scenes containing digital elevation models of the seafloor and digital terrain models of the topography, gas plumes exported from FMMidwater and Echoview^®^, ship’s navigation track lines, nautical charts, and other geographic information (Figure 5).

The automatic benthic chamber, Ada_N, developed in the frame of a collaborative agreement between RSE S.p.A. and the National Research Council of Italy funded by the Ministry of Economic Development, is a tool for measuring the flux of dissolved substances at the water-sediment interface. This is done through a multi-parameter probe and water sampling, analysed for nutrients, metals, DIC, dissolved gases, isotopes of the C and other dissolved pollutants [22]. In the pilot test, this equipment was used mainly as a collector of water samples.

**Figure 6:**
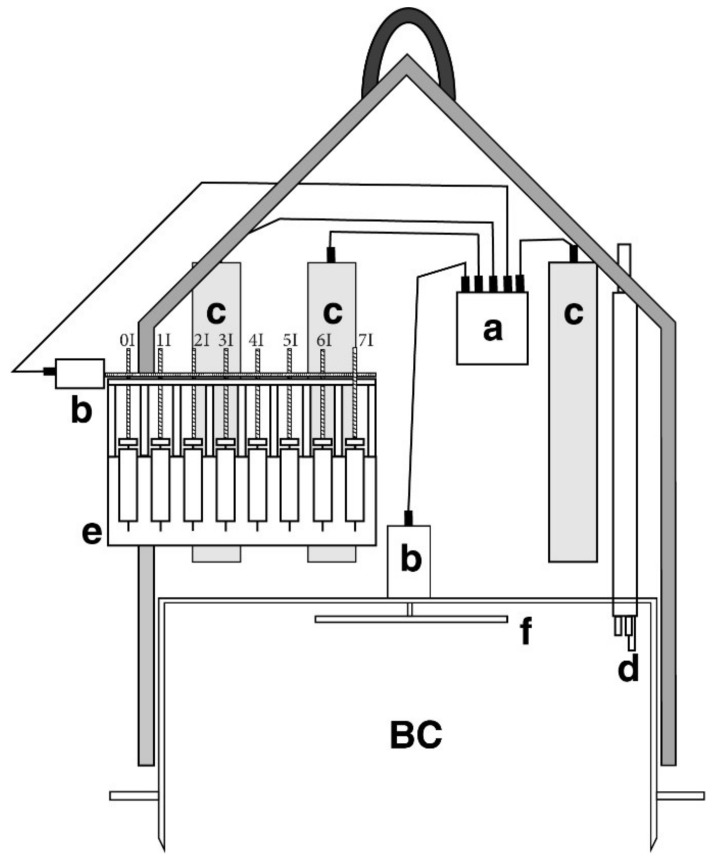
Schematic drawing of the Automatic Benthic chamber Ada_N. BC: measurement chamber. (**a**) electronic housing, (**b**) electric motor housings, (**c**) battery housings, (**d**) multi-parametric probe, (**e**) vampire system (sampling syringes), (**f**) rotating paddle. 0I to 7I are the vampire sampling syringes, syringe 2I is not a sampling device but is used to introduce a tracer inside the BC to measure its volume.

Ada_N is a Plexiglas cylinder open on the bottom and closed on top, which confines a known volume of water (approximately 100 L) overlying a known sediment area (3116 cm^2^) (Figure 6). Ada_N is equipped with an internal stirring system that reproduces the hydrodynamics near the seabed. The stirring system consists of a four-arm rotating paddle fitted on top of the inner side of the chamber. The paddle is actioned by the coupling of an electric motor with a neodymium magnet and turns at a speed of 4–6 rpm. Ada_N is also equipped with a multiparameter probe (Hydrolab MS5 from OTT HydroMet) for continuous monitoring of temperature, pH, conductivity, dissolved oxygen, Eh, and salinity (calculated) in the chamber. Furthermore, the vampire system collects water samples inside and outside the chamber and injects tracers inside the chamber at programmable times. The vampire is activated by an electric motor that initiates 8 syringes (Figure 6). The motors of the vampire and the stirrer are electronically controlled and supplied by three battery packs hosted in pressure compensated cases [22].

The benthic fluxes of each chemical are calculated considering the increase or decrease of concentrations inside the chamber during the deployment. The concentrations of each chemical inside the chamber are determined by measuring the concentrations in the water samples collected by the syringes or measured by the multi-parametric probe at different sequential times. The concentrations are then plotted against the sampling times and the slope of the regression line, calculated by a least-square fit, is determined. The benthic fluxes are then calculated by multiplying the slope of the regression line by the height of the BC. 

During the Spinaccia survey, Ada_N was deployed on the seabed at the Fontespina site at 11 m water depth for 9 h from morning to late afternoon, in order to evaluate if this device could sample liquid and gaseous substances related to hydrocarbon seepage during the pilot test. Furthermore, dissolved benthic fluxes were measured for total dissolved inorganic carbon (DIC), oxygen and pH, while salinity, temperature and depth were monitored all along the test. DIC content was measured by coulometric analysis with an in-house acidifier [23]. Oxygen, pH, salinity, temperature, and depth were measured by an inside chamber multi-parametric probe (Hydrolab MS5).

Water samples collected by Ada_N were refrigerated and transferred to the Ministry of Economic Development, DGS UNMIG Division V laboratories, where they went through a series of analyses that were re-designed on purpose for the pilot test:

1. Determination of gas compounds by headspace gas chromatography using a thermal conductivity detector (GC-TCD), model Agilent Technologies 7890A.
2. Measurement of the hydrocarbon index, following the ISO standard procedures UNI EN ISO 9377-2: 2002 [24] with a concentration above 0.1 mg/L. This technique involves the determination by gas chromatography with a flame ionization detector (GC-FID) of the extractable fraction related to hydrocarbons with retention times ranging between n-decane (C10H22) and n-tetracontane (C40H82) excluded.
3. Determination of metal composition by a Perkin Elmer OPTIMA 8000 inductively coupled plasma optical emission Spectrometer (ICP-OES) analysis using the APAT CNR–IRSA 3020 method [25].

For the determination of the gaseous compounds contained in the samples, 20 mL aliquots of water were transferred into vials with hermetic closure and heated in a water bath for about 1 h at 80 °C. Once the equilibrium between the aqueous and the gaseous phases of the sample was reached, the steam produced in the headspace was taken out with special syringes for GC (Hamilton 10 µL) and injected into the GC-TCD. The method of analysis included the following instrumental settings: column HP-PLOT PoraPLOT U, for allowing a more constant flux, symmetry of the chromatogram peaks and an overall higher-quality analysis of impurity in gas, solvent and hydrocarbons, set at –60–190 °C (30 m × 320 µm × 10 µm). Oven temperature 40 °C for 1.5 min 50 °C–150 °C at 10 °C/min. Inlet set at 150 °C, split ratio 5:1. Thermal conductivity detector (TCD) set at 250 °C. A known gas composition mixture was used, as a reference, for the screening of the seven sample’s headspace main components (such as oxygen, carbon dioxide, nitrogen, and methane). The analyses carried out for the evaluation of the gas content in water samples were mainly of a qualitative type, suitable for assessing the presence or absence of specific gaseous species.

The ICP-OES source consisted of a flowing stream of argon inductively (radio frequency field 40 MHz) ionized by a cooled coil surrounding a quartz “torch”, which supports and confines the plasma, at temperatures 6000–10,000 K. Data were processed with the Syngistix software. Elements’ typical wavelengths were scanned by the double-monochromator optical system achieving the quantification of linear dynamic ranges of four to six orders of magnitude for most elements. The analysis of metals concentration in water samples normally includes the preventive filtration and subsequent acidification of the solution with concentrated nitric acid up to pH ≤ 2, aimed to prevent or in any case delaying effects of precipitation, adsorption and even formation of metal complexes. Because of the issues described above, the best analysis procedure for the pilot test had to consider: high salinity of the water samples (about 35 g/L in NaCl), which required dilution; a rate of dilution not affecting the detection of low concentration metals. The best available technique was the use of a “simulated” matrix (salinity similar to sea water), used both for the preparation of the standards and “blank” solution, which reproduces the salinity of the diluted samples subsequently analyzed. The concentration of the metals is therefore calculated by building a calibration curve with a series of reference standards at different concentrations (Table 1). The simulated matrix was prepared with 32.9 g of NaCl in 1 L of bi-distilled water (typically 18.2 MΩ cm at 25 °C), then diluted 1:2 v/v and used for the preparation of both standard and blank solutions. The calibration curve was built on 3 points using a solution of NaCl (16.45 g/L) with multi-element standard solutions at different and increasing concentrations. 

The lowest concentrations of the curve were taken as a reference for determining the “Detection Limit” (D.L.) such as the lowest concentration of the analytes in a sample that can be detected in the method experimental conditions. Aliquots of about 20 mL of water samples were transferred to flasks and diluted 1:2 (v/v) with bi-distilled water; after they were acidified with a few drops of nitric acid up to pH ≤ 2. The accuracy and precision of the results were verified by analyzing standard reference solutions and the concentrations obtained were always within the 95% confidence interval of the certified values.

Marine sediments were collected with a small steel box corer in 12 stations, 6 for each site, including a reference site (REF in tables and figures) collected away from the seepages, midway between Fontespina and Bonaccia sampling sites. Each box core was subsampled at 2 cm intervals for the biogeochemical analyses down to a depth of 10 cm below the seafloor; XRF analyses were conducted on 7 samples selected on the basis of their spatial representability.

Marine sediments were analysed for the presence of pollutants, such as polycyclic aromatic hydrocarbons (PAHs). PAHs listed as priority pollutants were analyzed and these are listed as: naphthalene, (Nap); acenaphthene (Ace); Fluorene (Fl); phenanthrene (Phe); Anthracene (Ant); fluoranthene (Flu); pyrene (Pyr); benzo[a]anthracene (BaA); chrysene (Chr); benzo[b]fluoranthene (BbF); benzo[k]fluoranthene (BkF); benzo[a]pyrene (BaP); dibenzo[a,h]antracene (DahA); indeno[1,2,3,-cd]perylene+benzo[ghi]perylene (InP+BghiP). PAHs identification and quantification were performed using an HPLC system (Ultimate 3000, Thermo Scientific, Waltham, MA, USA) equipped with a fluorescence detector (RF-2000, Thermo Scientific

PAHs were extracted with dichloromethane:methanol (v/v) by ultrasonic bath (BRANSONIC 151 0E-MT) with three cycles of 20 min each. A liquid-liquid separation was carried out and the solution was initially concentrated on a rotary evaporator at 26 °C and then under a stream of N2. A hypersil Green PAH column (μm 2.1 × 150 mm, 1.8 μm, 120 Å) in a reversed-phase liquid chromatography with a water:acetonitrile (v/v) gradient elution was used. The mobile phase consisted of an initial composition of 60% acetonitrile (held for 6 min) that, after 15 min, reached 90% (held for 10 min) and then returned to initial conditions. The duration of the analysis was 31 min and the equilibrium time condition was 9 min. The flow rate was 0.3 mL min^−1^ at 40 °C. The wet weight (w.w.) of sediments was corrected to dry weight (d.w.), after determination of moisture in the samples. 

For the quality control, efficiency, and accuracy of the whole procedure, the external standard multipoint calibration technique was used to determine the linear response interval of the detector and the International Atomic Energy Agency (IAEA, Vienna, Austria) reference material, IAEA-408 and IAEA-383 were tested. The limit of detection (LOD) and the limit of quantification (LOQ) was calculated according to the standard ICH–5 A.D. [26].

Representative sediment samples from box cores were analysed at the Department BiGeA of the University of Bologna with a sequential wavelength dispersive X-ray Fluorescence (XRF) spectrometer (PANalytical AXIOS, the Netherlands), equipped with a 4 kW Rh tube and SuperQ 3.0 software. XRF analysis was used to determine the chemical composition of the sediments surrounding the seepage sites, and a reference sample from outside the study area helped for comparison. The total elemental chemistry was determined on thin-layer pressed powder pellet (Ф37 mm) in a boric acid binder, using 3 g of the dried and milled sample. 10 g of sediment samples were pre-treated with distilled water (Milli-Q©, Merck Millipore) at a liquid to solid ratio of 10:1, in order to remove the most soluble salts (Na, Cl) that would produce bias especially for accurate quantification of major elements. The samples were washed for 5 min in a centrifuge rotating at 3200 rpm. The number of washing cycles (approx. 15 cycles) was determined for each sample, reacting the supernatant recovered from the centrifuge vials with 10 mL of 1 mol AgNO_3_ until the formation of the white precipitate (AgCl) ceased. Total loss on ignition (LOI) was gravimetrically estimated after overnight heating at 950 °C, and the LOI values in percent were used for offline data correction. The following elements were analysed: Al, Ca, Fe, K, Mg, Mn, Na, P, Si, Ti (major elements, expressed in % wt., i.e., g/100 g, of the corresponding oxides) and As, Ba, Br, Ce, Cl, Co, Cr, Cu, Ga, La, Nb, Nd, Ni, Pb, Rb, S, Sc, Sm, Sr, Th, U, V, Y, Zn, Zr (expressed in mg/kg). The XRF calibration curves relied on a number of certified reference materials, including marine sediments; a certified reference material (TB, clay shale from ZGI) was measured as an unknown sample for quality control. The estimated precision for major and trace element determinations is better than 5% except for those elements at low concentrations (<10 mg/kg) where the precision is estimated 10–15%, and lower for halogens.

## 3. Results

### 3.1. Reflectivity of the Water Column and Sub-Seafloor Seismic Facies

**Figure 7:**
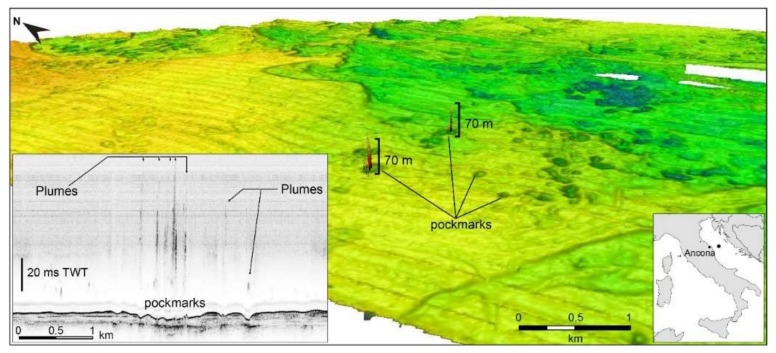
3D view of seafloor and water column reflectivity multibeam data in the Bonaccia area, some 30 nautical miles offshore Ancona (**lower right**). Up to 70-m-high gas plumes correspond to pockmarks on the seafloor and are visible also in the water column recorded by the CHIRP seismic profile (**lower left**).

**Figure 8:**
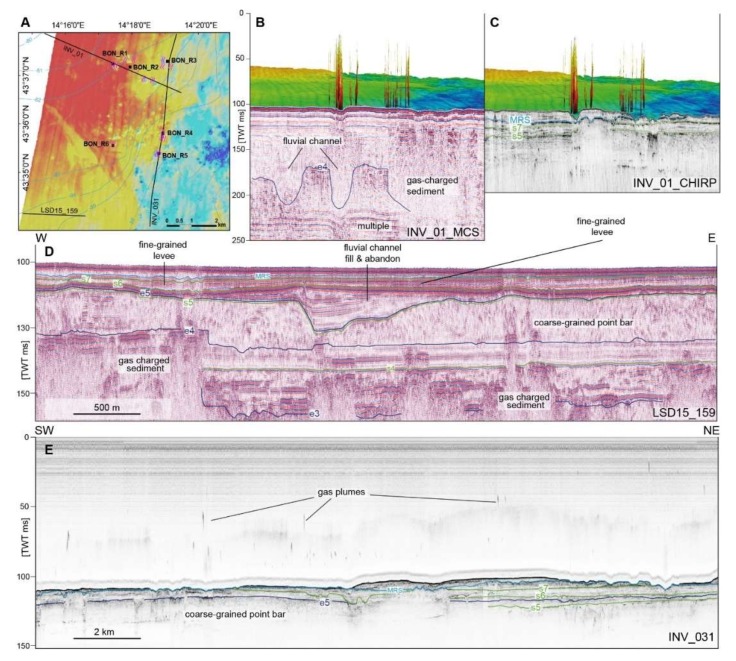
(**A**). Bathymetry map showing the seafloor corresponding to the last low stand Po alluvial plain, with location of the seismic profiles displayed in the panels, the box coring sampling stations, the location of the gas plumes detected with multibeam EM2040 in 2018 (see Figure 1 for symbol legend). (**B**). Multichannel seismic reflection profile INVAS_01 acquired in 2012 with superimposed gas plumes detected with multibeam EM2040 in 2018. (**C**). CHIRP seismic profile collected along the same track line of INVAS_01 with superimposed gas plumes detected with multibeam EM2040 in 2018. (**D**). Sparker seismic profile LSD15_159 acquired in 2015, showing the general seismic facies of the Po alluvial plain during the last sea level low stand. (**E**). CHIRP seismic profile INVAS_31 running beneath gas plumes detected with multibeam EM2040 in 2018. Isochrones: MRS = 14.4 ka (thousands years ago), s7 = 15.8 ka, s6 = 18.0 ka, e5 = 18.6 ka, s5 = 19.0 ka, e4 = 19.3 ka, e3 = 20.6 ka.

In the Fontespina site, about 24 km of multibeam lines were acquired for a total of 50 track lines and about 8 working hours. Data processing allowed to identify 31 plumes in the Bonaccia site and 110 plumes in the Fontespina site (Figure 5). For the Fontespina site, due to the density of gas plumes in the water column, an automated process using the Echoview^®^ software was preferred over a manual extraction, which is more precise but time-consuming. In the Bonaccia site, about 43 km of multibeam lines were acquired for a total 33 track lines and about seven working hours. The heights of the plumes in Bonaccia can reach up to 70 m from the seafloor (Figure 7 and Figure 8B,C) and here a more accurate manual extraction was preferred in order to locate the plumes in correspondence of bathymetry features, such as pockmarks and sub-seafloor seismic facies in the seismic reflection profiles (Figure 8).

From regional seismo-stratigraphic correlations [27,28] and based on previous seismic reflection profiles, we were able to trace to the study areas key stratigraphic horizons such as the MRS which corresponds to the maximum regression surface dated at 14.4 kyr cal before present (BP) and e3, dated at 20.6 kyr cal BP [27] (Figure 8C). These horizons, that correspond to isochrones, delimit sedimentary bodies comprising the channel-belt that deposited in the Po alluvial plain during the subaerial exposure related to the last glacial maximum (~22 kyr ago), when the sea level was lower than today by ~130 m (low stand phase) [29]. Such deposits are represented by coarse-grained point bars, buried below sparse channels and fine-grained beds that started developing during the first phases of sea level rise. Coarse-grained bodies are characterized by chaotic seismic facies which is indicative of coarse sediment charged with gas, while fine-grained sediments are characterized by laterally continuous and parallel reflections (Figure 8D). Below the coarse-grained bodies, transparent and laterally-discontinuous seismic facies may be indicative of gas-charged sediment, with fluids probably migrating from below (Figure 8D). The CHIRP profiles better show the uneven seabed characterized by seafloor depressions and incisions that might have developed in submarine conditions and that are in turn associated with gas plumes in the water column and the presence of fluid expulsion structures such as pockmarks in the bathymetric data (Figure 8E).

### 3.2. Biogeochemistry of the Water Samples

Data collected by Ada_N show that the benthic chamber worked at an average depth of about 11 m with weak variations due to tidal oscillations (11.04 ± 0.05), temperature during the deployment was steady (27.7 ± 0.01 °C) and represents the water column temperature of the bottom layer; also salinity show steady conditions (37.4 ± 0.01 °C). In the Fontespina site, fluxes of oxygen, dissolved inorganic carbon (DIC) and H+ (pH) were measured at the sediment-water interface by Ada_N (Table 2). Oxygen shows a constant decrease, corresponding to a medium flux of −51.53 mmol/m2*d. The negative oxygen flux is due to the microbic mineralization of the fresh organic matter deposited on the seabed.

The DIC fluxes show positive values of 42 mmol/m^2^*d (Table 2). Also, in this case, the positive flux is due to the aerobic and anaerobic mineralization of fresh and reactive organic matter deposited on the seabed before the experiment [30]. pH shows weak decreasing values around 8.20 (±0.007) corresponding to a flux towards the water column (or an increase inside the chamber) of 2e^−0.7^ H^+^ (Table 2). The pH decrease and subsequent H^+^ increase is a consequence of the DIC increase that acidifies the water inside the chamber. Moreover, the comparison of Fontespina oxygen and DIC flux with fluxes previously measured in Central Adriatic Sea highlights stronger positive (DIC) and negative (oxygen) fluxes in the Fontespina site (Table 2) [31]. 

Results of the hydrocarbon content analysis on seven water samples, collected with the benthic chamber in Fontespina, show that major gas compounds are: nitrogen, oxygen and only in some samples, traces of carbon dioxide and methane. C10–C40 content ranges between 2.03 to 36.9 mg/L, it appears that during acquisition from morning to afternoon the flux of hydrocarbons decreased (Table 3).

Regarding the metal composition, the results show a typical sea water composition and not specific metal species content increased in the water column (Table 4). 

### 3.3. Geochemistry of the Sediment Samples

**Figure 9:**
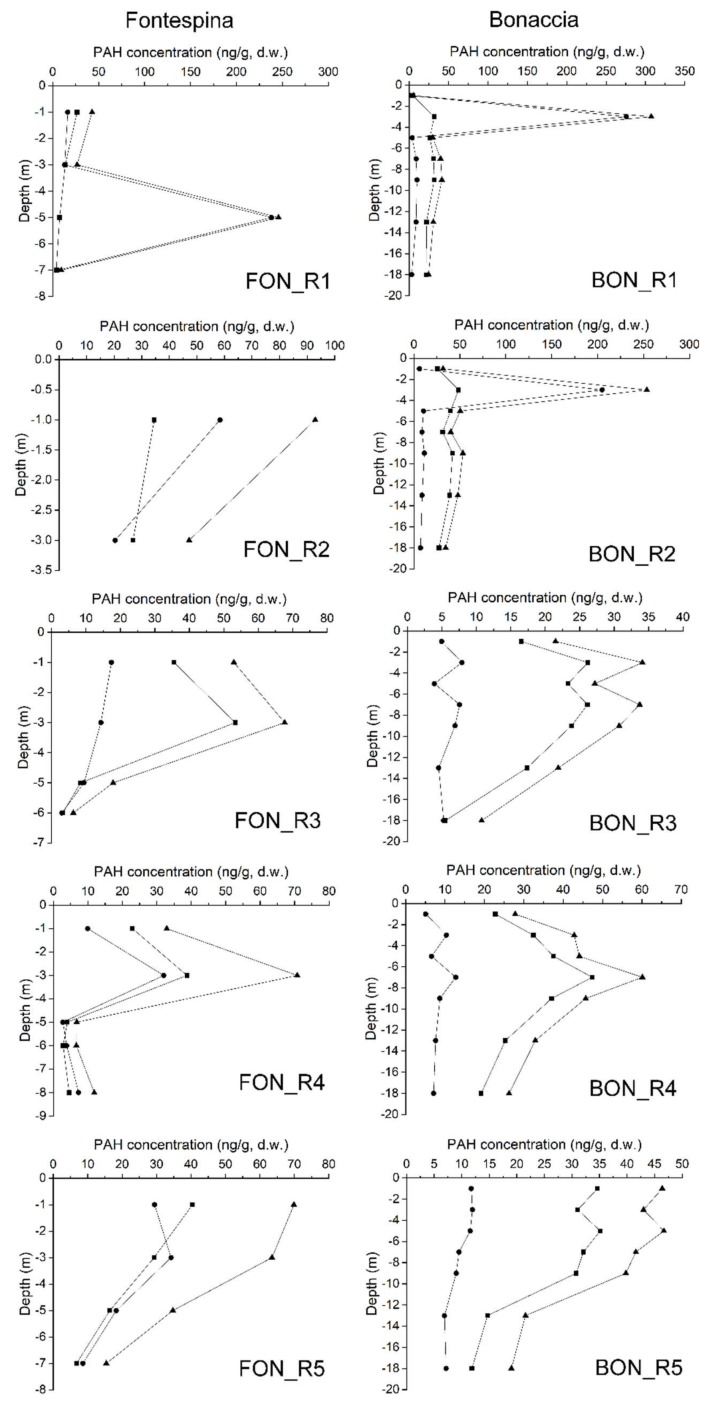
Total PAH concentrations (▲), PAHs with low molecular weight, LMW (•) and PAHs with high molecular weight, HMW (▪) recorded in the sediment layer of the Fontespina and Bonaccia sites.

**Figure 10:**
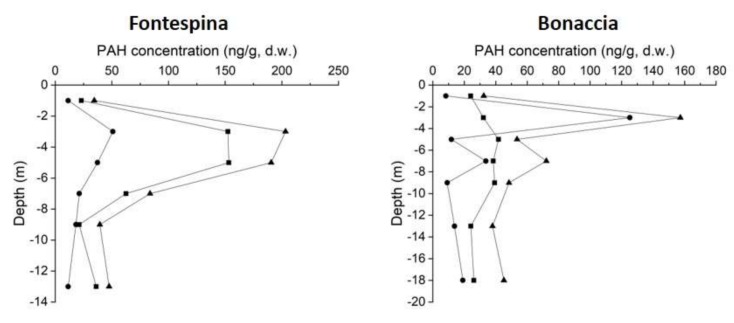
Total PAH concentrations (▲), PAHs with low molecular weight, LMW (•) and PAHs with high molecular weight, HMW (▪) recorded in the sediment layer of the reference site (REF) for Fontespina and Bonaccia sites.

**Figure 11:**
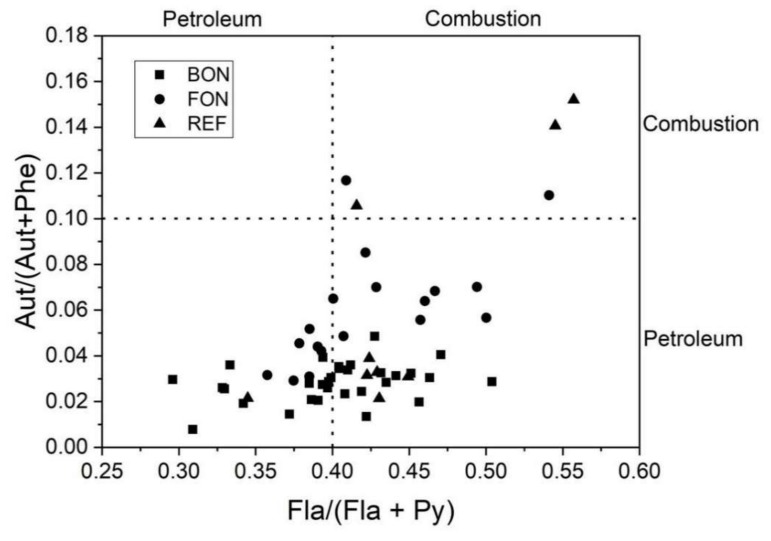
Cross plots of PAH diagnostic ratios Ant/(Phe+Ant) vs. (Fla/(Fla+Py) identifying sources of PAHs in all sediments layers of the Fontespina site (FON), the Bonaccia site (BON) and the reference site (REF).

Spatial and vertical distributions of PAHs were analyzed in the surface and subsurface sediment layers of both sampling sites and reference sites. Total PAH concentrations (ng/g, d.w.), low molecular weight (LMW) PAHs (sum of Nap, Ace, Fl, Phe and Ant) and high molecular weight (HMW) PAHs (sum of Flu, Pyr, BaA, Chr, BbF, BkF, BaP, DahA, InP and BghiP) were reported for each surface and subsurface sediment layers of Fontespina and Bonaccia (Figure 9) and reference site (Figure 10). Data show high variability of the PAH concentrations in the subsurface sediment layers with maximum values between −2 cm and −6 cm of depth below the seabed, for both sampling sites and reference site. Moreover, the Fontespina site recorded a LMW-PAH concentration greater than Bonaccia site, underling the dominant role of natural (biogenic and/or petrogenic) sources, which is related to the presence of the petroleum system which feeds the oil seepage. For a better understanding of the origin of investigated PAHs, PAH diagnostic ratios commonly used to identify the origin of PAHs in the environment were applied [32,33] (Figure 11). Levels of predominantly petrogenic PAHs were found in both sites, unlike the reference site collected away from the seepages, confirming the natural origin of these chemicals.

The XRF results show distinct chemical compositions for Bonaccia and Fontespina sites, respectively. While Si and Ca are high in both locations, Al is lower in Fontespina than in Bonaccia. The range of concentration of Cl, S (and Br) is increased in Fontespina, likely substantiating the hypothesis of higher levels of PAHs. Metals like Fe, Co, Ni, V, and Zn are comparably higher in Bonaccia suggesting potential mineralization taking place at the sediments surrounding the sampling site, which likely record a more complex diagenetic process. The LOI values average 21.8% (vs. 21.1% of the reference sample), signifying the apparent loss of volatile elements and total carbon during heating. Trace elements concentrations in both seepage sites were different from the reference sediment sample, a likely consequence of hydrocarbon contamination.

Comparing samples FON_R1 and BON_R3 collected at the sediment-water interface (Table 5), significant variation of extractable/exchangeable ions like Sr, Rb, Ce and V can be noted. 

## 4. Discussion

The measurements of dissolved benthic fluxes at the sediment-water interface are fundamental to better understand the marine biogeochemical cycles and alterations of aquatic ecosystems, as a consequence of human activities or natural processes [34]. Benthic fluxes are the dissolved chemical substances released or absorbed by the seabed as a result of early diagenesis processes or volcanic and hydrothermal/hydrocarbon release processes [35].

In aquatic sciences, benthic chambers have become a generally accepted approach for in situ measurements of fluxes of non-conservative elements that are involved in the biological and geochemical turnover of organic carbon, such as dissolved gases, nutrients, natural, and anthropogenic trace metals, and for in situ respiration measurements to estimate benthic carbon turnover. However, more recently, this approach has been also applied for organically enriched and biogeochemically active environments at continental margins such as mud volcanoes [36] and methane seep sites [37], where oxygen consumption can be extremely fast. However, in the latter studies, benthic chambers have been used specifically to monitor oxygen conditions inside the chambers or measure benthic fluxes from the enclosed bottom waters, however under no circumstances they were used before to sample and measure methane and other hydrocarbons in the water column. Chemical laboratories at DGS UNMIG Division V normally measure hydrocarbon content in water and air samples, collected from emissions of offshore platforms, in the frame of the Italian national inspection mechanism of oil and gas activities. In this collaboration, the laboratories have re-designed existing analytical procedures to the new type of water samples for determining gas composition by headspace analysis and metal traces by modifying the procedure usually applied with ICP technique. Overall the procedure proved successful, the water samples collected by the benthic chamber were suitable and contained enough hydrocarbons to complete the analysis. 

We used conventional studies of benthic fluxes on data acquired with the benthic chamber to try and infer on the biogeochemical cycles associated with the seepage in Fontespina. The differences of DIC and oxygen fluxes between the two sampling areas (Bonaccia and Fontespina, Table 2) with similar early diagenesis processes suggest the presence of greater contents of local, fresh and reactive organic matter and/or older and thermogenic organic substances possibly due to hydrocarbons seepage contributions. This supports the capability of the benthic chamber system to record the contribution of hydrocarbon seeps to sediment-water fluxes. The hydrocarbons indeed undergo partial mineralization that consumes oxygen and produces an excess of DIC flowing upwards. 

The test carried out in the Fontespina site shows that the comparison between dissolved fluxes measured in an affected seepage site and in a blank site is able to detect the presence of the seepage and diagenetic components of the benthic fluxes and their contribution. For these reasons, the benthic chamber may be considered a promising tool to evaluate the contribution of the hydrocarbon seeps to the local dissolved benthic fluxes. 

Furthermore, the benthic flux measurements of additional chemicals, such as carbon isotopes, nutrients, Fe and Mn, and the pore water analyses (O_2_, NO_3_, Fe, Mn, SO_4_, DIC, NH_4_, and PO_4_) would have given information on the biogeochemical processes in the first centimeters of the sediment that are involved in the early diagenesis processes. In such a way, the respective contribution of early diagenesis and deeper-seated processes to the hydrocarbon seepages can be discerned [35]. 

PAHs concentrations in the sediments and diagnostic ratios allowed to assess the natural origin of the hydrocarbons in both sampling sites, ruling out an anthropogenic component. In addition, in the seabed layer of the two study areas, the average of the total PAH concentrations of five samples out of 6 (R1, R2, R3, R4, and R5 in Figure 9) were higher in Fontespina (58.3 ng g^−1^) compared to Bonaccia (26.5 ng g^−1^). This finding is of particular importance for the monitoring of seepage located close to the coasts, where they may interfere with tourism and leisure activities, and being of concern for public health. Indeed, along the Fontespina beaches, bathers complain about episodes of oil discharge. The cause is often searched in a ship’s or a nearby offshore plant’s spill, but most probably the oil comes from the seepage area characterized by ethane and propane discharge (Table 3 and [17]), which are photochemical pollutants, in the water, while the sediments around the seep are naturally enriched in PAHs. The new integrated tool proposed here could be used to implement a monitoring program, with periodical sampling of water and sediments near the seep, to achieve a predictive model of seep intensity and seasonality (we observed that, likely, the intensity of the discharge is higher in the morning) and design a warning system with social and touristic benefits. 

We also tried to use the XRF results on sediments to establish some predictive tools to better understand the origin of the seep, in terms of source depth. The XRF data collected normalized to Upper Continental Crust (UCC) values show enrichments in Ca, Mg, and P and a significant depletion in the element Fe, which is prone to changes of redox conditions in marine environments. For normalization to North American Shale Composite (NASC) values [38], the pattern is similar (data not shown) having the negative anomalies of Al, Ti, K, and Si slightly more elevated, except for P, which adheres to the shale averages.

**Figure 12:**
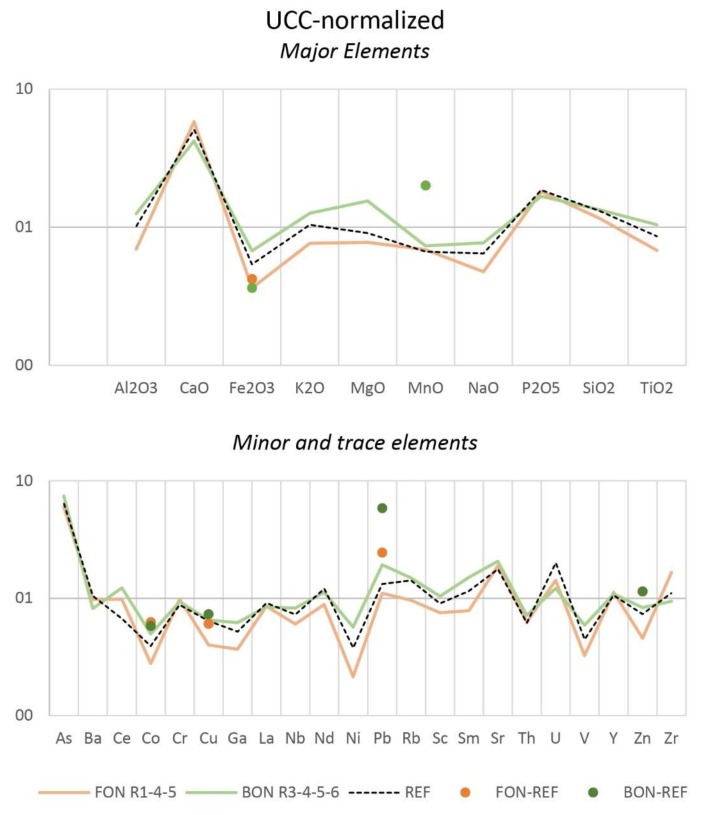
Average values of Fontespina (orange) and Bonaccia (green) sites and their reference sample (REF) normalized to Upper Continental Crust (UCC) according to [39]. Reference data points for both sites averaged sampling stations 1, 40, 76 (for Fontespina site, “REF” orange dots) and 13, 33, 85 (for Bonaccia site, “REF”, green dots) reported elsewhere [40].

Ni, V, Co and other chalcophile elements are depleted to the UCC due to their tendency to form sulphides whereas Pb concentrations likely enhanced as a result of high mineralization towards deeper water depth and reducing conditions (Figure 12). In the Fontespina site, the hydrothermal component is visible from the anomaly peaks of the elements typically associated with ultramafic rocks and further corroborated by the relatively high Ti and Cr confronted to NASC averages. However, according to the distance of anomaly peaks between the two trends, the hydrothermal component has a much higher impact at the Bonaccia site. 

**Figure 13:**
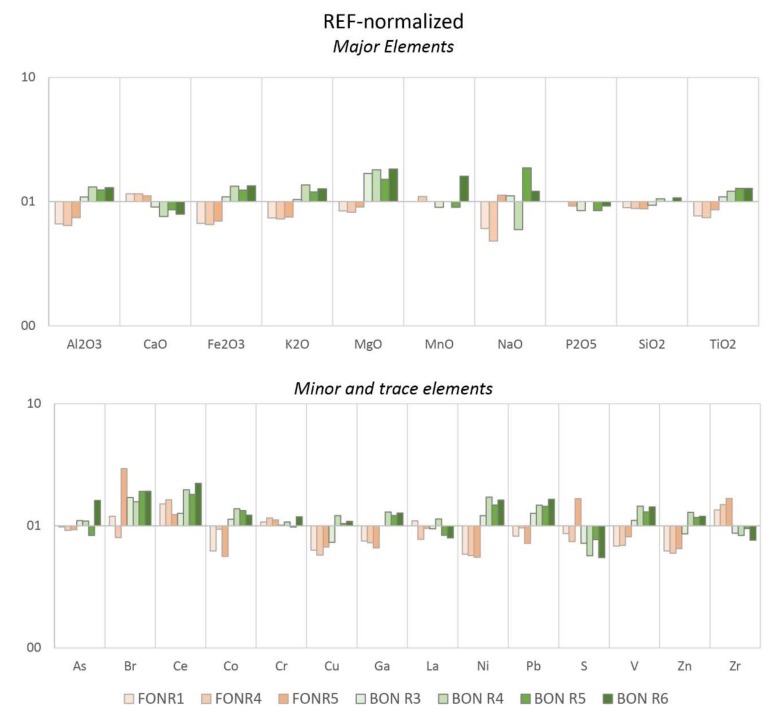
Enrichment factors of selected elements derived from the reference sample (REF) of this study. Orange and green histograms display the calculated ratios for Fontespina and Bonaccia sites, respectively.

The hydrothermal Mn/Fe and Cu/Fe ratios increase in the sediments collected from Bonaccia site compared to the reference site (Figure 13). These ratios for sample BON_R3 are close to the Fontespina trends, suggesting a gradual decrease of the hydrothermal component to coastal sediments. The sample BON_R6 shows a marked Mn enrichment (Figure 13): this likely pertains to deposits formed by early diagenesis in deep-sea sediments. Absolute concentrations of Fe, Mn, Ti, Cu and Zn are also much higher at Bonaccia than Fontespina, suggesting that a relatively higher-temperature plumbing system impacted the chemical composition of the sediments at this sampling site. Total V, Pb and As concentrations are strongly enhanced in the Bonaccia samples compared to Fontespina. 

The Ce/Ce* ratios calculated from the NASC-normalized content of the samples as Ce/Ce* = 2·Ce/(0.67·La + 0.33·Nd) and Ce_anomaly_, as log(Ce/Ce*), helped to highlight the variation of redox-sensitive metals [41]. A positive Ce anomaly is found ranging from 0.26 to 0.43 with slightly higher values for the sediments of Bonaccia site. When confronted the reference sample REF (Ce_anomaly_ = 0.07) a significant variation of Ce anomaly is seen at both sites that can relate to a reductive environment as to further confirm that the samples used for comparison are hydrogenous or diagenetic. Following the method by [42], we estimated the source components for each element, [X], derived from [X]_total_ = [X]_detritial_ + [X]_ultramafic_ + [X]_hydrothermal_ + [X]_biogenic_, where [X]_total_ is the measured concentration by XRF and [X]_biogenic_ is an adjunctive component calculated as [X]_biogenic_ = [X]_total_ − [X·P_NASC_/LOI_REF_·P_sample_/LOI_sample_]. A positive hydrothermal contribution is thus estimated for elements precipitated directly from vent fluids or scavenged from seawater like Ti, As, Co, Sm, and Zn. As an average, we valued as the biogenic contribution a fraction of 3.5–4% of the total weight. The P/Al ratio is considered a sensitive indicator of biogenic contribution; for this dataset, the P/Al ratios are around 0.02, similar to those reported for turbidites where chemostratigraphy of the Quaternary deposits was attempted [43], and higher than average shale (<0.01).

Further insight into the diagenetic processes and rock-sourcing shall be obtained from isotopic analysis that will be carried out as a next step, so that these findings should be taken cautiously; however overall we infer that a deeper-sourced fluid contribution (i.e., from carbonatic rocks below the Pleistocene biogenic pools) to the petroleum system can be suggested. Indeed, in the Central Adriatic Sea, the last glacial eustatic cycles, which led several times to subaerial exposure due to relative sea level fall and erosion, favoured the formation of biogenic gas in the finer and richer organic matter layers, intertwined with sands, which became large reservoirs now exploited for hydrocarbon production. In particular, in the Pleistocene (starting around 1800 ka), a full set of regressive sandy/clay beds was deposited during a relative sea level fall. In the basal sandy levels of this cycle, several biogenic gas pools formed at the current depth of 1200 ms (TWT), where hydrocarbon traps are represented by gentle anticlines, draping an old tilted block of the Adriatic margin, as in the case of the Bonaccia field [44]. Methane-derived authigenic carbonates were collected at the seafloor in the Bonaccia area and are believed to have formed during the Late Pleistocene sea-level oscillations, even though the origin of the gas, that favoured their precipitation, remains elusive [20]. Commercial-scale multichannel seismic profiles do not have sufficient resolution and sometimes fail to display subtle features that may confirm a direct link between shallower manifestations such as seafloor pockmarks, carbon precipitation products and water column plumes with the deep hydrocarbon reservoir and even deeper strata, such as the underlying Mesozoic carbonates. On the contrary, in shallower sedimentary layers, our seismic reflection profiles have enhanced resolution and allow to precisely placing the gas plumes in the water column on top of sub-seafloor seismic facies and features (Figure 8). The water column reflectivity and sub-seafloor seismic reflections show where seeps are preferentially distributed and, ultimately, where leakage from a reservoir can occur, considering that seeps may act as fluid escape pathways over prolonged periods of time, contributing to reservoir depletion [45]. From our data, coarse-grained sedimentary bodies seem to favour the accumulation and trapping of hydrocarbons, especially when sealed by fine-grained sediments such as levee deposits, belonging to the fluvial system developed during the sea level low stand. When a channel incises the coarse-grained bodies, this becomes a preferential pathway of fluid conduit along which seepage concentrates, and as a result, pockmark and gas plumes are found preferentially on top of channel beds (Figure 8). Our seismic data show only the last glacial-eustatic cycle, however we suggest that a similar pattern of seepage distribution can also be found in the Early Pleistocene coarse-grained layers of the hydrocarbon reservoir and that the hydrocarbon source is deeply seated below these biogenic pools and has a thermogenic contribution. 

## 5. Conclusions

Surface geochemical exploration and hydro-acoustic methods combined are effective in detecting and measuring seeps on the seafloor and sub-seafloor and gas plumes in the water column. Real time measurements at gas and methane seepage sites provide geophysical, benthic fluxes and chemical measurements taken directly from the source. This new multidisciplinary and multi-instrumental method proved to be effective for fast detection of water column anomalies and effective in sediment and water sampling for hydrocarbon content and other geochemical proxies. 

The benthic chamber Ada_N is a promising tool to sample hydrocarbon seeps and to evaluate their contribution to the local dissolved benthic fluxes. Additionally, dissolved benthic fluxes could be supported by the study of the early diagenesis processes. These are able to explain the genesis of the fluxes and the role of other chemicals, such as trace elements and organic matter components. A more exhaustive set of chemical analysis such as carbon isotopic composition, as well as nutrients, Fe and Mn, would give a more comprehensive characterization of the hydrocarbon seep environment. 

On the whole, the combination of surface geophysical, geochemical and sedimentological methods proposed in this study has proved to be an excellent way to localize and characterize bottom seepages. The use of this technology is easy and fast to set-up, cost-effective since it may bypass the employment of divers, as well as effortlessly relocatable because it can be mounted on a small vessel. The only current restriction is the operational water depth limit to 100 m that is overcome by the employment of larger instrumentation like the AMERIGO lander [22]. 

Immediate applications of this system include:

- Monitoring of coastal areas, which might be critical when continuously shifting water dynamics due to seasonality and other factors can limit and modify vertical methane and oil migration affecting the variable intensity of discharge on the coast (e.g., Fontespina oil spill).
- Monitoring leakage from abandoned or decommissioned wells and sealines.
- Detecting reservoir leakage through fractures and faults, without expensive 3D seismic acquisitions or tailored re-processing of seismic data.
- Fast detection of upward migration of biogenic methane along the boreholes, originating for example, from shallow gas accumulations that are penetrated when drilling into the underlying deep hydrocarbon reservoir (e.g., [46]).
- De-risking oil & gas exploration by using economic and cost-saving surface probing methods, rather than expensive 3D exploration seismic programs.

Future directions of this application include the geochemical investigation of different types of minerals that precipitate around hydrocarbon seeps. In particular, seeps can be associated to metal-bearing deposits of commercial interest, such as Fe–Mn oxyhydroxide and carbonates. Precipitation in sub-surface sediments leads to quantitative adsorption of dissolved rare earth elements (REE) from the fluid plume and enrichments in the surrounding sediments [47] and accumulation of baryte [48,49], which has been recently added to the list of critical raw materials for the EU. Furthermore, specific microbial communities are indicative of the presence of hydrocarbons in the seabed sediments and microbial prospecting has been used for decades for petroleum exploration [50]. Since relatively extremophile bacteria can be expelled from subsurface via seepage, advanced metagenomic approaches would allow the characterization of the microbial signature, indicative of the characteristics of the deep petroleum system, and complementing the oil genetic fingerprint that can also be achieved with PAHs specific compound ratios [51]. 

## Figures and Tables

**Table 1:** Standard Calibration Curve Concentrations for metal composition analysis of water samples.

Calibration Curve (ICP)			
Metals	Point 1 (ppm)	Point 2 (ppm)	Point 3 (ppm)
STD 21 (Cd, Cr, Cu, Pb, Mn, As, Be, Se, Co, Ni, Zn, V)	0.01	0.1	0.5
Fe, B, Ba, Al	0.1	1	10
Sn	0.01	0.1	0.5

**Table 2:** Oxygen, DIC and H+ dissolved benthic fluxes measured by Ada_N in Fontespina and on pelitic sediments of the Central Adriatic Sea.

Dissolved Benthic Fluxes (mmol/m2*d)			
Site	Oxygen	DIC	H+
Fontespina	−51.53	42	2e^−0.7^
Central Adriatic Sea ^1^	-39	9.38	n.d.

^1^ average of fluxes measured in summer and fall on the pelitic Holocene wedge between Ancona and S. Benedetto del Tronto, which mimic the Bonaccia site conditions [30].

**Table 3:** Hydrocarbon content (C10–C40) in the water samples.

C10–C40	
Sample	mg/L
0 I	28.6
1 I	36.9
3 I	<L.R
4 I	0.56
5 I	2.47
6 I	2.03
7 I	<L.R

**Table 4:** Metal concentrations in water samples at Fontespina (mg/L). D.L. detection limit. Accuracy within the 95% confidence interval of the certified values.

Values (mg/L)							
Metal Species	0 I	1 I	3 I	4 I	5 I	6 I	7 I
Al	<D.L.	<D.L.	*<D.L.*	<D.L.	<D.L.	<D.L.	<D.L.
Cd	<D.L.	<D.L.	<D.L.	<D.L.	<D.L.	<D.L.	<D.L.
Cr	<D.L.	<D.L.	<D.L.	<D.L.	<D.L.	<D.L.	<D.L.
Cu	<D.L.	<D.L.	<D.L.	<D.L.	<D.L.	<D.L.	<D.L.
Pb	<D.L.	<D.L.	0.0468	<D.L.	0.0190	0.0228	<D.L.
Mn	<D.L.	<D.L.	<D.L.	<D.L.	<D.L.	<D.L.	<D.L.
As	<D.L.	<D.L.	<D.L.	<D.L.	<D.L.	<D.L.	<D.L.
B	4.1744	5.3180	4.5720	3.8472	5.2658	5.3828	4.1482
Ba	<D.L.	<D.L.	<D.L.	<D.L.	<D.L.	<D.L.	<D.L.
Be	<D.L.	<D.L.	<D.L.	<D.L.	<D.L.	<D.L.	<D.L.
Co	<D.L.	<D.L.	<D.L.	<D.L.	<D.L.	<D.L.	<D.L.
Fe	<D.L.	<D.L.	<D.L.	<D.L.	<D.L.	<D.L.	<D.L.
Ni	<D.L.	<D.L.	<D.L.	<D.L.	<D.L.	<D.L.	<D.L.
Se	<D.L.	<D.L.	<D.L.	<D.L.	<D.L.	<D.L.	<D.L.
Sn	0.0636	0.0632	0.0632	0.0632	0.0632	0.0632	0.0632

**Table 5:** Major and trace elements of the sediment samples collected at Fontespina and Bonaccia sites (FON, BON). REF stands for reference site representing the average conditions of the Central Adriatic sediments in use for both sites.

Element	REF	FON_R1	FON_R4	FON_R5	BON_R3	BON_R4	BON_R5	BON_R6
**wt.%**								
Al_2_O_3_	8.24	5.46	5.29	6.12	8.98	10.81	10.22	10.67
CaO	25.41	29.34	29.37	28.22	22.99	19.31	21.85	20.14
Fe_2_O_3_	3.36	2.25	2.2	2.34	3.67	4.47	4.15	4.5
K_2_O	1.56	1.15	1.13	1.17	1.62	2.13	1.87	1.98
MgO	2.57	2.16	2.11	2.33	4.33	4.64	3.89	4.7
MnO	0.1	0.1	0.11	0.1	0.09	0.1	0.09	0.16
NaO	1.58	0.96	0.76	1.78	1.76	0.94	2.95	1.91
P_2_O_5_	0.13	0.13	0.13	0.12	0.11	0.13	0.11	0.12
SiO_2_	36.89	33	32.46	32.25	34.54	38.95	36.97	39.55
TiO_2_	0.43	0.33	0.32	0.37	0.47	0.52	0.55	0.55
LOI	21.08	22.18	22.18	22.38	22.98	20.87	21.82	20.39
**mg/kg**								
As	9.6	9.4	8.8	8.9	10.6	10.5	8	15.5
Ba	277	253	267	258	188	240	215	224
Bi	0.4	0.5	0.4	0.5	0.5	0.5	0.5	0.5
Br	15.1	18.1	12.1	44.4	25.7	23.8	28.9	29
Ce	26.9	40.7	43.9	33.2	34	53.2	48.8	59.8
Cl	4948	3880	2015	9224	4828	1716	8378	4724
Co	9.8	6.1	9.2	5.5	11.1	13.5	13.1	12
Cr	158	170	183	177	160	170	155	187
Cu	16	10.1	9.2	10.7	11.7	19.4	16.7	17.4
Ga	8.8	6.6	6.4	5.8	8.8	11.4	10.7	11.2
La	18.2	20	14.1	17.3	17.2	20.7	15.2	14.5
Nb	8	6.4	6.7	6.8	8.1	9.5	9.7	9
Nd	24.1	19.2	20.3	13.5	11.2	24	25	29.7
Ni	39.6	23.2	22.6	21.9	47.9	68	58.6	64.3
Pb	13.2	10.9	12.7	9.5	16.7	19.5	19.1	21.8
Rb	70.9	47.7	47.4	48.6	58.8	80.7	78.5	79.8
S	1047	904	778	1747	752	596	809	574
Sc	12.6	15.7	5.5	10.4	1	17.6	23.1	16.5
Sm	4.6	3.3	3.2	2.9	5	6.5	6.2	6.4
Sr	424	462	453	446	547	453	527	451
Th	3.5	4	3.3	3.4	2.7	3.4	5.3	4.9
U	3	2.7	1.5	2.2	1.4	1.5	2	2.4
V	86.9	59.2	60.1	70.7	96.3	126	113	124
Y	19.1	20.1	20.3	20.1	18.2	20.5	20.9	18.8
Zn	52.1	32.5	31.1	33.9	44.9	67.2	61.2	62.5
Zr	138	186	206	231	120	115	131	105
